# ‘Building back better’ or sustaining the unsustainable? The climate impacts of Bank of England QE in the Covid-19 pandemic

**DOI:** 10.1057/s41293-022-00223-w

**Published:** 2023-01-03

**Authors:** Daniel Bailey

**Affiliations:** Manchester Metropolitan University, Manchester, M15 6BH UK

**Keywords:** Bank of England, Climate change, Net zero, Quantitative easing, Green finance

## Abstract

The environmental impacts of monetary policy received academic attention after the 2008 financial crisis and the ‘market neutral’ quantitative easing policies that followed. This article examines the Bank of England’s Corporate Covid Financing Facility (CCFF) and the Asset Purchasing Facility (APF) between June 2020 and June 2021 to assess whether the Bank’s response to the COVID-19 pandemic was aligned with the transition to sustainability. The data indicates that the Bank of England’s monetary allocation schemes again served as a panacea for businesses with ecologically intensive business models and a Treasury committed to restoring the pre-existing growth model. Indeed, the Bank’s QE schemes now represents an element of the crisis management governance that repeatedly ‘locks in’ the ecologically-calamitous economic trajectory at potential critical junctures. The Bank’s shielding of its technocratic and depoliticised status has thus far inhibited any leadership role in tackling the climate crisis, despite its growing power as an actor of economic governance at times of crisis and purported enthusiasm to ‘build back better’.

## Introduction

Current financial patterns are deeply implicated in the climate crisis due to the tendency of the financial markets to sponsor infrastructure and systems of production that generate greenhouse gas emissions and deforestation, most egregiously in the aviation, automotive, fossil fuel energy and agricultural sectors (Newell 2019). Transforming these financial patterns is imperative for any transition to environmental sustainability. Central banks play an increasingly powerful and direct role in either bolstering or subverting the financial patterns underpinning path-dependent economic trajectories at times of crisis (Ryan-Collins 2013; Matikainen et al. 2017; Campiglio et al. 2018) and as such are pivotal actors in any state-led sustainability transition (Duit et al. 2016; Bailey 2018; Newell 2019; Eckersley 2020).

Quantitative Easing (QE), which entails the purchasing of financial assets by central banks, epitomises the growing power of central banks. Previous QE schemes have been criticised for a commitment on behalf of central bankers to ‘market neutrality’; i.e. the tendency to conform to the investment preferences of the capital markets in order to minimise the impact of the purchases on the relative prices of financial assets. This market-conforming guiding principle has been seen as an attempt to depoliticise the growing power of unelected central banks in order to protect their technocratic legitimacy and operational independence from elected bodies (Tucker 2018; Klooster and Fontan 2019; Dӧnmez and Zemandl 2019). Since the 2008 financial crash, market neutrality has fortified the pre-existing economic status quo and, consequentially, its environmental tendencies (Ryan-Collins 2013; Matikainen et al. 2017; Campiglio et al. 2018; Dafermos et al. 2018). The pandemic-induced economic downturn of 2020 evoked further rounds of QE, and some called for states to ‘build back better’—including the Governor of the Bank of England (henceforth, the Bank) (Bailey et al. 2020)—through a policy response that addressed climate change and other socio-economic issues.

This article interrogates the capital allocated by the Bank of England’s QE schemes and the consequences of its allocative decisions on the trajectory and environmental impacts of the UK economy. The Bank’s official data on the usage of the Corporate Covid Financing Facility (CCFF) and the Asset Purchasing Facility (APF) between June 2020 and June 2021 are examined to identify the support extended to the organisations and sectors most associated with greenhouse gas emissions and deforestation, and thereby the climate impacts of the Bank’s governance in the Covid-19 crisis.

The findings reveal that the Bank of England’s QE policies served to shore up the ecologically unsustainable economic status quo at a potentially pivotal moment of critical juncture. The Bank’s continued fear of distorting the monetary allocations of the capital markets when administering QE schemes replicated the structural bias towards industry incumbents, with significant consequences for the UK economy’s ecological footprint in the post-pandemic period. Despite the Bank’s recognition of the climate risks to financial stability and its institutional remit to address such threats, the failure of capital markets to price in climate risks was once again effectively been mirrored by the Bank. The composition of QE purchases, combined with the absence of any ‘green’ conditionality, ensured the survival of companies with carbon-intensive business models. Covert monetary financing of Treasury expenditure meanwhile created the space for a green fiscal stimulus, but Treasury action on decarbonisation through crisis management measures was equally negligible. As such, crisis management monetary policies helped ‘lock in’ an ecologically-catastrophic economic model, and constituted a lethal blow to the possibility of ‘building back better’.

This article will begin by outlining how the Bank’s institutional mandates and norms have historically conditioned the mobilisation of QE finance and the environmental ramifications of these policies. It will then scrutinise the Bank’s 2020 QE asset purchases and the impacts of its allocative choices on the sectors and economic model accountable for present levels of greenhouse gas emissions. Finally, it will discuss the politics of ‘greening’ the Bank and its role in a sustainability transition.

## The Bank of England’s ‘market neutral’ QE pre-pandemic

Whilst the state is a potentially pivotal agent of decarbonisation, and economic crises tend to prompt interventions that could serve as steering opportunities, the state is certainly no *tabula rasa* (Johnstone and Newell 2018). The Bank’s actions response to climate change and the consequences for its governance are—as with all state agencies—conditioned by institutional objectives, mandates (or interpretations of those mandates), policy tools, resources, and leadership. An institutionalist analysis of the idiosyncratic remits and evolution of state agencies is, therefore, crucial to comprehending the character of governance strategies and the prospective role of central banks (and the state more broadly) in sustainability transitions.

The UK’s central bank has professed a monetarist inflation-targeting mandate since the stagflation of the 1970s (Ingham 1984; King and Katz 2021), when it jettisoned strategic credit targeting and broader economic objectives in order to eschew the ‘financial repression’ of unfettered capital markets (Bezemer et al. 2018). However, in the context of fiscal austerity of the 2010s, the Bank of England undertook an extraordinarily active and experimental monetary policy regime that seems to belie the purported focus on ensuring price stability and upholding trust in the value of Sterling. The base interest rate was lowered repeatedly to encourage bank lending and consumer spending, but the most startling innovation has been Quantitative Easing. QE schemes entail the creation of central bank reserves ex nihilo for the purpose of purchasing financial assets, in order to increase liquidity both directly and indirectly via increasing demand and reducing the yield and cost of borrowing for the issuers of bonds. Launched in successive rounds, and mirrored by other central banks around the world, the Bank’s QE schemes has financed a series of large-scale Treasury and commercial bond purchases designed to shore up the fiscal position of the state and encourage investment in the core economy.

Thus far, the asset purchases comprising QE has largely conformed to the investment preferences of the capital markets. A disinclination to distort market activity has engendered this principle of ‘market neutrality’. As the Bank’s Monetary Policy Committee (2017) put it, the ‘intention has been to minimise interference in the private sector credit allocation process by buying a portfolio which is representative of issuance by firms making a material contribution to the UK economy’.

Market neutrality has been located in the desire to depoliticise the growing power of Central Banks in order to maintain technocratic legitimacy and operational independence from elected bodies (Best 2016; Tucker 2018; Papadia and Välimäki 2018; Dӧnmez and Zemandl 2019; Klooster 2022). Following Klooster and Fontan (2019), “central bankers… are openly concerned that the use of unconventional tools threatens their independence (Group of Thirty 2015; Goodhart and Lastra 2018)” and “central bankers, accordingly, try to counteract repoliticisation and these efforts shape their policies”. This creates dual processes of politicisation and depoliticisation in central banking (Sørensen and Torving 2017). Klooster and Fontan (2019) argue that “it is through market neutrality that “central bankers keep decisions on new monetary instruments in the domain of their expert judgment, and thereby outside the domain of democratic politics” (Klooster and Fontan 2019). Depoliticisation itself can be understood as a strategic mode of statecraft designed to enhance the power of agents in governance institutions to implement unpopular or politically difficult decisions or policies (Burnham 2014). Depoliticisation discourses are typically employed to justify political actions that shore up prevailing economic growth models (Berry and Lavery 2017, Dӧnmez and Zemandl 2019). From this perspective, depoliticised market neutrality can be seen as indicative of the power wielded by vested interests (Ingham 1984; Burnham 2014).

By affirming the tendencies of the capital markets, the principle of market neutrality underpinning QE has had environmental impacts. The unwillingness of financial markets to penalise unsustainable industrial activities and turn to low-carbon investment (Gabor et al. 2019; Shrivastava et al. 2019; HM Government 2019) have been mirrored by ‘market neutral’ QE, despite the Bank’s attempts to encourage markets to ‘price in’ climate risks. This means that the pre-existing UK growth model, with its prevailing patterns of rising inequality and ecological degradation, has effectively been reinforced at moments of crisis by the Bank’s structural bias towards industry incumbents when making allocative decisions (Ryan-Collins 2013; Adolph 2013; Green and Lavery 2015; Volz 2017; Klooster and Fontan 2019). Since 2009, QE has persistently served to ‘lock in’ economic systems that generate various patterns of ecological degradation (Unruh 2000).

A study by Matikainen et al. (2017) found that 49.2% of the Bank’s corporate bond purchases by March 2017 were concentrated in the manufacturing and electricity production sectors responsible for 52% of the UK’s GHG emissions. The European Central Bank (ECB) reflected this tendency, with 62.1% of bond purchases focused on the manufacturing and electricity and gas production sectors responsible for 58.5% of GHG emissions in the Eurozone. Renewable energy companies, holding a relatively minor position on the bond market, were entirely overlooked by both the Bank’s and ECB’s QE schemes. The authors concluded that ‘the carbon-intensive skew of these purchases raises concerns of disproportionately increasing prices and encouraging additional debt issuance in high-carbon relative to low-carbon sectors’ (Matikainen et al. 2017, p. 1).

Reinforcing these investment tendencies may be depicted as neutral insofar as its avoids distorting existing markets but, insofar as such a depiction can ever be true (Klooster and Fontan 2019), market-neutral QE can certainly not be seen as ‘neutral’ in social or environmental terms (Matikainen et al. 2017; Gabor et al. 2018, 2019). This prompted calls to redesign QE. Some called for the exclusion of assets with significant climate risk in future QE schemes (NGFS 2018; Gabor et al. 2018), whilst others have more ambitiously called for ‘Green QE’ which entails financing a low-carbon transition with the monetary purchasing of green bonds (Lucas 2011; Ryan-Collins 2013; Bailey and Craig 2018; Dafermos et al. 2018; Gabor et al. 2019).

In March 2020, as the severity of the COVID-19 pandemic became clear and the government announced a de facto supply-side shutdown of non-essential businesses, the Bank announced further rounds of monetary stimulus. The Bank’s allocative choices in the 2020 pandemic would inescapably inflate certain asset prices in ways which would have long-lasting effects on the character of the UK economy’s recovery and its ecological footprint.

There were reasons to think that the Bank would reform its crisis management monetary policy operations. There was a growing recognition that the financial sector was perpetuating the industrial operations generating climate change and the Bank had demonstrated awareness that climate risks represented systemic threats to financial stability that could potentially be germane to the macroprudential remit. This appeared to underscore the dangers of repeating policies that replicated the financial market’s failure to ‘price in’ climate risks, and alternative QE schemes had been widely deliberated (FT 2014; Dafermos et al. 2018; Campiglio et al. 2018; Gabor et al. 2019). Meanwhile central banks, and states more broadly, had increasingly being identified as a culprit of the ‘climate emergency’—at least in part because of the activism of new social forces including the ‘School Strikers’, Extinction Rebellion and divestment campaigns—and there was growing support for ‘greener’ forms of economic governance (Ipsos Mori 2020). As such, the Covid-19 crisis represented a potential inflection point; a crisis that permits challenges to pre-existing dogmas, policy tools and objectives (Blyth 2002). The experimentation following the 2008 financial crash suggested that the Bank were not excessively bound to a path-dependent trajectory (Eichengreen et al. 2011; Goodhart et al. 2014), and there was a rhetorical commitment from the current and former Governors of the Bank to ‘build back better’ (Bailey et al. 2020). The confluence of circumstances prompted speculation about the possibility of radical changes to UK economic governance.

## ‘Lock in’ during lockdown: the climate impacts of the CCFF in the midst of Covid-19

The Covid-19 pandemic triggered a series of state interventions in to economy and, as with the 2008 financial crisis, the Bank played a prominent crisis management role. This invoked questions about how aligned the Bank of England’s quantitative easing schemes would be with the purported sustainability transition. To what extent, therefore, has QE allowed us to—in the words of Andrew Bailey and Mark Carney (Bailey et al. 2020)—‘build back better’?

The Bank of England’s portfolio of purchases, comprising the Quantitative Easing scheme, escalated to £895bn in the pandemic; equivalent to over 40% of annual UK GDP (BoE 2020a). The CCFF was designed to provide liquidity to ‘eligible businesses…making a material contribution to the UK economy’ through the purchase of ‘short-term debt’ (BoE 2020b). Its intention was to help businesses pay wages and suppliers throughout the Covid-19 related disruptions to cash-flow. Purchases were financed by the creation of central bank reserves, and provided on terms comparable to those prevailing in markets before the pandemic, with the Treasury backstopping the loans (BoE 2020b). This section will examine the eligibility criteria for the CCFF and the sectoral composition of the assets purchased to ascertain the ‘green credentials’ of the Bank’s QE crisis response and its contribution (or not) to a green economic transformation.

The CCFF eligibility criteria set out by the Bank foreshadowed the limited change in the composition of QE asset purchases. The emphasis on making a ‘material contribution to economic activity in the UK’ and ‘investment grade’ credit quality as of March 1st 2020 is explicable but also hinted at the continued structural bias to incumbent market actors. The debts of companies with business models predicated upon atmospheric degradation were also *not* excluded from the scheme, and indeed were well positioned given the standing criteria, which suggested that the recognition of climate risks had not permeated this area of the Bank’s operations.

The eligibility criteria established the framework in which 11.62% of QE liquidity was channelled towards the aviation sector, 10.13% towards the automotive sector, and 6.47% toward the energy sector. These sectors—particularly complicit in generating greenhouse gases—collectively acquired 28.22% of the CCFF. The construction, manufacturing, retail and finance sectors—each requiring some forms of transformation to meeting decarbonisation targets—were also financial supported through the pandemic by this scheme.
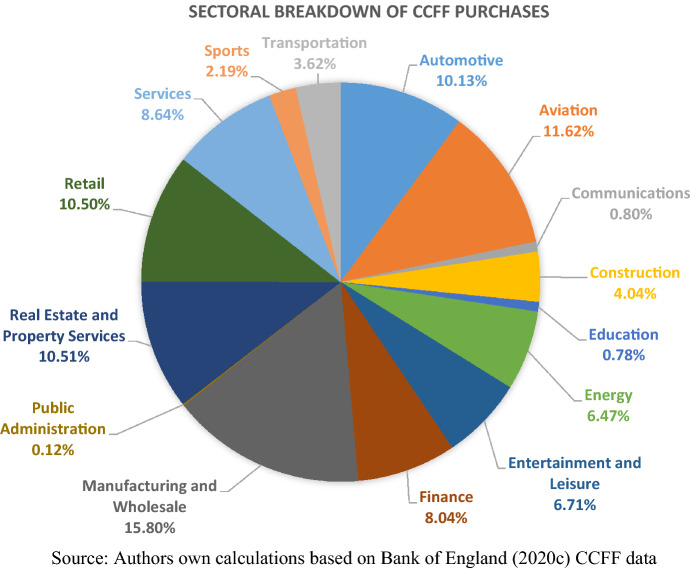


A closer examination of the companies receiving QE liquidity illustrates the unsustainable character of the business models being supported. Aviation companies British Airways, Ryanair, EasyJet, Jet2, Airbus, Gatwick Airport, Flight Centre UK, and Whizz Air collectively benefitted from £2.89bn of QE finance to help them survive the economic uncertainty and are thus well set for the continuation of ‘business as usual’ in the post-pandemic period. In the automotive sector, companies including Rolls Royce, Honda, Alliance, Mitsubishi, Paccar, Inchape, Toyota and Nissan benefitted from £2.52bn. Petrofac, Baker Hughes and Schlumberger in the oil and gas energy sector benefitted from £1.315bn. In addition, the Bank’s finance assisted Chemring Group and Meggit Plc (providers of components, technology and services for the aerospace and defence industries) to the value of £210 mn, Amcor (manufacturers of paper packaging) to £360 mn, ICL Group (agricultural chemicals) to £50 mn, and BASF (manufacturer and wholesale retailer of chemicals) to £1bn. None of these companies can boast sustainable business models, yet liquidity to the cumulative value of £8.345bn was provided to these firms without ‘green strings’ attached.

These sectors supported by these measures are deeply complicit in the climate crisis. The energy sector alone is estimated to emit 24% of total industrial greenhouse gas emissions in the global economy, which is compounded by its contribution to the emissions of households and commercial properties (17.5%) and the emissions related to energy production (5.8%). The automotive sector meanwhile (cars, trucks, lorries, motorcycles and buses) is responsible for 11.9% of total emissions, and the aviation sector responsible for 3.5% (Our World in Data 2016).

In contrast, the firms with business models that promote the decarbonisation of the UK economy are under-represented in the scheme. Only Iberdrola International and Worley Energy**,** recipients of £100 mn and £195 mn respectively, have renewable energy operations (as well as significant fossil fuel energy and petrochemical manufacturing operations). Meanwhile the £30 mn of liquidity provided to the National Trust will help shore up an existing system of land management that offsets GHG emissions. £325 mn, however, is only 1.3% of the resources mobilised as part of this scheme.

The economic effects of these policy decisions elude quantification due to the multitude of interacting causal factors affecting corporate share prices, the diverging timeframes of the support extended and the CCFF data available, and the absence of a counterfactual that would offer a comparison. Certainly, as acknowledged by the House of Lords Economic Affairs Committee, the Bank’s actions lowered bond yields for beneficiary companies and it is likely that the Bank’s monetary support may have restored confidence in these companies during the crisis period and generated a ‘crowding in’ effect across the bond and stock markets (House of Lords 2021).

The Bank’s 2020 QE scheme, therefore, again favours market incumbents in spite of the climate risks pertaining to business models. The dilemma between tackling short-term pandemic-related financial instability has seemingly been prioritised over tackling medium-term climate-related financial instabilities. This is entirely intelligible given the potential scale of financial instability presented by the pandemic and subsequent supply side shutdown, but the disregard of climate risks (which threaten to be even greater) should be of great concern in the aftermath. The Bank’s structural priorities, and the influence market incumbents are able to exert over the Bank’s governance (Braun and Gabor 2019), will once again ensure the financial viability of (and, thus, ‘lock in’) economic systems that will wreak ecological devastation (Unruh 2000). Indeed, during economic crises particularly, QE now represents a decisive element of the co-evolutionary interactions between governing institutions, market activities and technological systems that serve to repeatedly entrench ecologically-calamitous economic trajectories (Unruh 2000). UK QE has become a panacea for major corporate polluters at times of crisis.

An extenuating factor is the shortage of AAA-rated bonds in low-carbon industries. Even if the Bank were to prioritise ‘green bonds’ they would be relatively scarce. A green investment bank operating under government direction and subject to government guarantees would certainly help provide more, and there would be a transformative effect on the economy, but this is beyond the Bank’s mandate. Excluding firms with unsustainable business models appeared a more plausible scenario, but supporting actually existing businesses and employment was prioritised above climate goals. This places the Bank far behind other central banks in aligning monetary policy operations with environmental imperatives (Reclaim Finance 2021).

The current absence of an agreed taxonomy of investment purchases (Gabor et al. 2019) problematises the analysis of the Bank’s investment choices, but the bias to carbon-intensive market incumbents is evident; echoing prior rounds of QE (Maitkanen et al. 2017). The Bank’s favouring of the aviation industry, the automotive sector, and the fossil fuel energy sector profoundly undermines the purported desire to ‘build back better’.

## The asset purchasing facility and monetary financing of fiscal policy during Covid-19

The Bank’s QE programme was not limited to the CCFF in the Covid-19 pandemic. The Bank’s APF also purchased Treasury bonds on the secondary market as part of the crisis response. Indeed, the vast majority of these purchases were of UK government debt. The Bank’s three rounds of QE in 2020 raised the total amount of government debt owned by the Bank by £450bn; from £425bn to £875bn (BoE 2021b; House of Lords 2021). This means, remarkably, that the British state owns an increasing share of its own debt. Indeed, the value of the Treasury bonds on the Bank’s books now surpasses that of overseas investors and pension and insurance companies (FT 2021). This serves to assist the government’s huge programme of borrowing by keeping debt servicing costs low. This de facto bolstering of state capacity has been in operation, to differing extents, since 2009.

Governor Andrew Bailey is keen to proclaim that this does not represent the monetary financing of government expenditure (Bailey 2020a, b). Indeed, this may not be the primary motivation of the Bank either, despite the perception of the majority of leading actors in the UK government bond market (FT 2021), with the bond purchases also serving to improve the functioning of the gilt market and counteract a tightening of monetary and financial conditions (BoE 2020c, d). As Gabor (2021) notes, the strategic objective of ensuring private sector financing conditions and anchoring inflation expectations means that it can be thought of as ‘shadow monetary financing’ rather than the subordinate form of monetary financing that was common in the post-war period. However, enlarging the fiscal capacity of the state is unavoidably a side effect of these asset purchases. The Bank’s purchases have kept pace with Treasury issuance, which have kept gilt yields in check (House of Lords 2021; FT 2021). The blurred division between monetary and fiscal policy has seemingly become a valuable aspect of contemporary crisis management. Monetary financing of government debt—whether strategic or a byproduct of other objectives—has challenged previous depictions of state capacity; i.e. that the state’s expenditure and activities is dependent on the ‘tax take’ generated by the private sector and the money borrowed from capital markets through bond auctions in order to fund its various programmes. Operationalising QE in this way, in combination with the Bank’s ‘Ways and Means’ facility which provides the Treasury with an effectively unlimited overdraft capacity (without which the government may have struggled to remain solvent in March 2020 according to Andrew Bailey (2020b), has fundamentally expanded the capacity of the state. This is relevant because the enlargement of state capacity opened the fiscal space for large-scale investment in a green economic transformation, if the government possessed the inclination (Coppola 2019). It cannot be ignored that supporting fiscal expenditure in this way theoretically offered the Bank an alternative and vital (albeit supportive) role in inducing a state-led sustainability transition. These benefits are precisely why fiscal and monetary coordination, of the type evident today, has been deemed an institutional precondition for a dirigiste ‘Green State’ (Bailey 2018).

This fiscal space was ultimately not utilised by the government to finance a green Keynesian project in the Covid-19 crisis. Despite the apparent enthusiasm by some in the Bank for democratic state to lead the sustainability transition with fiscal instruments (Bailey et al. 2020), fiscal policy-makers did not capitalise on this new era of monetary financing to fund green Keynesian projects. With fiscal and monetary resources both being directed at preserving the economic status quo rather than ‘green’ investments, the Treasury appear equally focused on perfecting the tools of crisis management to restore the prevailing growth model than coordinating path-shaping economic transformations.

## The politics of ‘greening’ the Bank of England: incremental institutional evolution during a climate emergency

The current investment decisions made in the financial sector continue to be implicated in the ecologically calamitous economic status quo whilst decarbonisation deadlines become ever tighter and the catastrophic manifestations of climate change become increasingly visible (Gabor et al. 2019; NGFS 2019; Bolton et al. 2020). Macroeconomic modelling exercises project numerous possible scenarios, depending on the scale climate change and the character of political responses, but current investment patterns will beget ecological degradation and thus extensive financial and macroeconomic instability (Batten et al. 2016; Dafermos et al. 2018; Lamperti et al. 2019). This signals the need for a swift transition of the financial sector’s investment portfolios, guided by central banks, to ensure financial stability. A sustainability transition requires a central bank that accounts for systemic climate risks, even if this necessitates bucking market trends (Gabor et al. 2019; Robins et al. 2021). This not only includes QE that precludes unsustainable business models, but also the issuing of green sovereign bonds, modifying the eligibility criteria for its collateral framework, incorporating the climate risks of collateral assets in the lending and derivative markets, and obligating firm and institutional investors to disclose the climate impacts and risks of their activities (Gabor et al. 2019; NEF 2020). It also includes safeguarding the state’s capacity to orchestrate economic investment in low-carbon economic activity despite GDP levels (Bailey 2018; Eckersley 2020).

Central banks vary greatly across the world in terms of institutional mandates, objectives, norms and exposure to the lobbying efforts of powerful vested interests. This renders some more predisposed to ecological protection (amongst other forms of action) than others. The Bank of England appears particularly averse to adopting an overtly political or ‘distortive’ path-shaping role due to fears about undermining its cherished technocratic status and operational independence (Bailey 2020a), albeit it may potentially support the decarbonisation projects of more democratic state agencies overseeing fiscal policy. The continued market-conforming approach to QE in the pandemic locks in the UK economy’s path-dependent trajectory and undermines attempts to meet decarbonisation targets.

Bank officials are not ignorant of the systemic risks of climate change. Mark Carney made several proclamations on the ‘climate risks’ to financial stability (Carney 2017, 2019; BoE 2019); which incorporates both the risks of physical risks of climate-related extreme weather events (e.g. droughts, floods, and storms) and longer-term gradual changes in the climate (e.g. sea level increase, changes in rainfall), and the transition risks arising from technological innovations, changing consumer preferences or political action intended to aid decarbonisation (Zenghelis and Stern 2016). Precise calculations are beset by contestation over variables such as future technological and market-based innovations and fiscal policy trends, but there is little doubt that direct property damage and disruption to business operations, indirect financial and operational impacts from disruptions to the operations of suppliers or consumers, decreased agricultural production capacity, and increased insurance claims and liabilities could all have a considerable impact on economic and financial instability (NGFS 2019; Bolton et al. 2020). The threat remains that current patterns constitute a ‘carbon bubble’ which would ‘pop’ in systemically destabilising ways if carbon-intensive political economies were to take seriously their legally binding carbon reduction targets and demand existing fossil fuel assets were left in the ground (McGlade and Ekins 2015). Mark Carney has expressed fears of changing ecological conditions prompting a ‘climate-driven Minsky moment’—a sudden collapse in asset prices which ripples throughout the financial system and global economy (Carney 2019)—and has urged financial markets to transition towards more sustainable investments.

These risks could potentially have influenced the Bank’s asset purchasing decisions, as part of its ‘macroprudential’ remit to assess, monitor and address systemic financial risks. The policy consequences of this remit remain subject to fierce contestation (Baker 2015; Hungin and James 2019), but the discernible institutional change has thus far been limited to petitioning financial institutions to disclose their exposure to climate risks and introducing climate-related ‘stress testing’ in the hope that engendering ‘perfect information’ will ‘naturally’ disincentive carbon-intensive investment. As such, the remit has not thus far profoundly affected QE crisis management. The prospect of excluding fossil fuel assets from the Bank’s future bond purchases and altering the composition of the Bank’s asset portfolio was described as a ‘very strong argument’ by Andrew Bailey in a Treasury Select Committee in March 2020, where he claimed that he intended to make it ‘a priority’ (Clarke 2020). This would also dovetail with the call made by the ‘Network for Greening the Financial System’ (NGFS)—a network co-established by Carney when Bank Governor alongside representatives of eight other central banks and financial regulators (NGFS 2018, 2019)—for central banks to ‘integrate sustainability into their own portfolio management’ (BoE 2019); calls already heeded by central banks elsewhere in Europe (DNB 2017; Norges Bank 2017). Some have gone further and called for ‘Green QE’—the modification of the eligibility criteria of asset purchases in order to mobilise resources to low-carbon economic activity or a national Green Investment Bank—which Carney accepted was a possibility in correspondence with Caroline Lucas (FT 2014; Dafermos et al. 2018; Campiglio et al. 2018; Bailey and Craig 2018; Gabor et al. 2019). Yet the Bank’s QE programme has seemingly remained unaltered by the macroprudential remit and the increasingly urgent need to address the climate crisis, just as it has seemingly ignored the criticisms of the social and distributional tendencies of previous QE schemes (Ryan-Collins 2013; Green and Lavery 2015; Matikainen et al. 2017; Campiglio et al. 2018) and its limited success in increasing investment and lending (Ryan-Collins 2013; Haldane et al. 2016; House of Lords 2021). By channelling resources to the oil and gas energy, aviation and automation industries without conditionality, the recent bout of QE served to rescue forms of production that exacerbate ecological degradation and amplify climate risks.

The Bank’s desire to render QE ‘market neutral’, and work alongside (rather than confront) the financial markets, signifies the Bank’s desire to retain its apolitical technocratic status at a time when its power was increasingly evident. Dual dynamics of politicisation and depoliticisation are once again notable here in the attempts to shore up the dominant national growth model (Sørensen and Torving 2017; Berry and Lavery 2017; Klooster and Fontan 2019). These tendencies have significantly curbed ambitions of leadership and authoritative steering on transformation despite growing awareness of climate and financial instability.

Institutional evolution has been incremental at a time when the transition to sustainability must be rapid. As the NGFS note, meeting decarbonisation targets requires ‘a massive reallocation of capital’ (BoE 2019), yet the incremental policy changes made by the Bank over recent years in the context of ever greater climate urgency and the unsustainable investment preferences of the financial sector seem to foreshadow either climate-related Minsky moments or climate breakdown. Indeed, the incrementalism and market-conforming tendencies of the Bank of England seem to suggest a disavowal of a significant role for central banking in the ‘rapid, far-reaching and unprecedented changes’ demanded by the IPCC to achieve 45% GHGs reductions by 2030 (IPCC 2018). Despite the apparent enthusiasm for ‘building back better’ within the Bank (Bailey et al. 2020), there is no desire for the Bank to play a direct role as ‘builder’.

This is not to say that monetary policies ought to be considered primary tools for instigating long-term structural change. QE is not, in isolation at least, well-designed for orchestrating structural economic transformation, which would require a far more comprehensive suite of policies and systemic changes of which monetary policies represent only one component (see Gabor et al. 2019; Robins et al. 2021). Yet central banks do have a pivotal role in realigning global finance with the risks posed by climate change, and in this instance the Bank has formed part of the salvo of state policies intended to uphold the inhibit rather than promote structural change through stabilising the economic status quo at a potentially path-shaping juncture (Berry et al. 2022). Senior Bank actors have expressed a preference to ‘build back better’ (Bailey et al. 2020), but responsibility for ‘building’ was eschewed in this economic recovery.

This leaves the Bank of England a relative laggard in this area of central banking. Despite its reputation as a ‘early mover’ and primary advocate on climate mitigation amongst comparable organisations due to its role in the NGFS and developing climate risk analytics, the ECB, De Nederlandsche Bank and the Banque de France amongst others have made far greater strides in greening monetary policy operations (Dikau and Volz 2021; Siderius 2022; Klooster 2022). ECB governors have already accepted that “market neutrality may not be the appropriate benchmark for a central bank when the market by itself is not achieving efficient outcomes” (ECB 2020) and has pledged to account for climate change in its core policy decisions and “adjust the framework guiding the allocation of corporate bond purchases to incorporate climate change criteria” (ECB 2021).

The existing politics of the Bank, however, may potentially be affected by the Treasury’s bestowal of a new mandate to account for the climate impacts of bond issuers, announced in March 2021, so that the Bank plays a more active role in the transition to a ‘net zero’ economy (BoE 2021a). This institutional development, and indeed the broader ecological context of climate change, could act as a catalyst for challenges to the commitment to market neutrality in future monetary policies and the Bank’s existing asset portfolio. After the new objective was announced, the Bank publically committed to undertake a review of its Corporate Bond Purchase Scheme to ‘account for the climate impact of the issuers of the bonds’ (BoE 2021c), and later committed to incrementally ‘tilting’ their portfolio away from carbon-intense firms (BoE 2021d). This potential institutional turn from market neutrality to economic transition could theoretically help steer the financial sector towards decarbonisation. Yet, there is justifiably scepticism that the Bank’s approach will substantially alter environmental impacts of future asset purchases, given that its approach will continue to reflect the existing sectoral composition of bond markets even if holdings will be tilted within those sectors; an indication that market neutrality will continue to shape its actions on climate risk (Dafermos et al. 2022).

Moreover, inflationary pressures have accelerated a programme of ‘quantitative tapering’ that quashes prospects of imminent large-scale asset purchases. The next round of QE, even if infused with the objective to mitigate climate risks to financial instability, may occur too late to facilitate an economic transformation that meets decarbonisation targets.

## Conclusions

The empirical findings reveals that the Bank’s resources were mobilised to shore up the pre-existing economic model at a time of potential critical juncture; thereby ‘locking in’ an economic trajectory characterised by escalating ecological degradation and climate risks. This round of QE thus failed to break from the established principle of market neutrality (Ryan-Collins 2013; Green and Lavery 2015; Matikainen et al. 2017; Campiglio et al. 2018; Dafermos et al. 2018), despite the growing awareness of climate risks to financial stability and the changes to the Bank’s mandate.

QE does not in isolation represent a perfectly designed tool for orchestrating structural economic transformation, but has in this instance formed part of the salvo of state policies intended to uphold the economic status quo at a time of potential path-shaping change (Berry et al. 2022). Senior Bank actors expressed a preference to ‘build back better’ (Bailey et al. 2020), but responsibility for ‘building’ was eschewed in this economic recovery.

These market-conforming allocative tendencies are in part borne of a desire to shield the Bank’s depoliticised technocratic status with which its operational independence is bound up, which results in measures that support pre-existing economic structures. This strategy, however, spawns a host of distinctly *political* and invidious distributional and ecological consequences. Indeed, it could contribute to a seismic market failure that ultimately threatens to undermine the Bank’s claim to be an apolitical institution. This prompts the question: how far into the climate crisis will the Bank travel before its notional conflation of institutional legitimisation with ‘market neutral’ governance is weakened by the ecological ramifications of perpetually fortifying the carbon-intensive status quo? The recent expansion of the Bank’s remit to facilitate the transition to ‘net zero’ may prompt some degree of institutional evolution (subject to the political conflicts over the interpretation of the new remit), and it is possible that the foundations of central bank legitimacy will shift considerably in the era of climate catastrophe.

Nonetheless, the Bank’s extant current economic governance, and the political ideas enshrined in its *modus operandi,* breed pessimism that the Bank will feature in any putative state-led sustainability transition. Not only is the Bank’s evolution insufficiently radical to play a role in a sustainability transition on the timescales demanded by IPCC forecasts (IPCC 2018), but it now appears that its increasing power is part of the British state’s crisis management strategy at times of crisis to shore up the economic status quo and quell any prospect of transformative change.

## Appendix

**Table:**

Businesses with Commercial Paper held by the CCFF between June 2020 and April 2021, categorised by economic sector	Nominal value of Commercial Paper held by the CCFF at highest point, in £mns	Sectoral % of resources deployed
Automotive		
Alliance Automotive Investment Ltd	20	
Honda Finance Europe Plc	185	
Honda Motor Europe Ltd	480	
Inchcape Plc	100	
Mitsubishi Corporation Finance Plc	300	
Nissan Motor Co. Ltd	600	
PACCAR Financial Plc	170	
Rolls-Royce Plc	300	
Toyota Financial Services (UK) Plc	365	
	2520	10.13228258
Aviation		
Airbus SE	500	
British Airways Plc (International Airways Group Plc)	300	
EasyJet Plc	600	
Flight Centre UK Ltd	115	
Gatwick Airport Ltd	275	
Jet2 Plc	200	
Ryanair DAC	600	
Wizz Air	300	
	2890	11.61995899
Communications		
Telefónica Europe B.V	200	
	200	0.804149411
Construction		
ACS Actividades de Construcción y Servicios S.A	50	
Etex NV	55	
J.C.B. Service	600	
Lendlease Europe Finance Plc	300	
	1005	4.04085079
Education		
London Business School	50	
London School of Economics & Political Science	80	
Roehampton University	5	
University of Leicester	60	
	195	0.784045676
Energy		
Iberdrola International B.V	100	
Petrofac Ltd	300	
Schlumberger Plc	415	
TechnipFMC Plc	600	
Worley UK Finance Sub Ltd	195	
	1610	6.473402758
Entertainment and Leisure		
Ansco Arena Ltd	45	
Bourne Leisure Ltd	300	
Carnival Plc	25	
Fuller Smith & Turner Plc	100	
Greene King Ltd	300	
Intercontinental Hotels Group	600	
RCL Cruises Ltd	300	
	1670	6.714647582
Finance		
ABB Finance B.V	400	
Baker Hughes UK Funding Company Plc	600	
DXC Capital Funding DAC	600	
ITOCHU Treasury Centre Europe Plc	100	
SSP Financing Ltd	300	
	2000	8.04149411
Manufacturing and Wholesale		
Akzo Nobel NV	30	
Amcor UK Finance Plc	360	
BASF SE	1000	
Bayer AG	600	
CNH Industrial N.V	600	
Goodwin Plc	30	
ICL Group Ltd	50	
Johnson Controls International Plc	370	
Meggitt Plc	160	
Molson Coors Brewing Company (UK) Ltd	1	
Orbia Advance Corporation SAB de CV	300	
Polypipe Group Plc	100	
The Vitec Group Plc	50	
Unternehmensgruppe Theo Müller S.e.c.s	50	
Vesuvius Plc	200	
Young & Co.’s Brewery Plc	30	
	3931	15.80153593
Public Administration		
The National Trust for Places of Historic Interest or Natural Beauty	30	
	30	0.120622412
Real Estate and Property Services		
Anchor Hanover Group	140	
Aster Treasury Plc	100	
Flagship Housing Group Ltd	50	
Hammerson Plc	75	
London & Quadrant Housing Trust	300	
Notting Hill Genesis	300	
OPTIVO	150	
Paragon Asra Housing Ltd	75	
Peabody Trust	100	
Places for People Ltd	150	
Platform Housing Group Ltd	100	
Sanctuary Treasury Ltd	300	
Thames Valley Housing Association	175	
Westfield UK & Europe Financial Plc	600	
	2615	10.51425355
Retail		
ASOS Plc	100	
Burberry Ltd	300	
Chanel Ltd	600	
Greggs Plc	150	
John Lewis Plc	300	
Kingfisher Plc	600	
Marks and Spencer Plc	260	
The Boots Company Plc	300	
	2610	10.49817056
Services		
Brake Bros Ltd	600	
Chemring Group Plc	50	
Compass Group Plc	600	
G4S International Finance Plc	300	
Rentokil Initial Plc	600	
	2150	8.644606168
Sports		
Football Association Ltd	175	
Football League Ltd (The)	75	
The Arsenal Football Club Plc	120	
Tottenham Hotspur Stadium Ltd	175	
	545	2.191307145
Transportation		
FirstGroup Plc	300	
National Express Group Plc	300	
Stagecoach Group Plc	300	
	900	3.618672349
